# Smart glasses for monitoring vital signs in anaesthesia care settings: a qualitative simulation study

**DOI:** 10.1186/s12871-025-03501-4

**Published:** 2025-12-01

**Authors:** Per Enlöf, Carina Sjöberg, Mona Ringdal, Sophie Lindgren, Axel Wolf, Pether Jildenstål

**Affiliations:** 1 Institute of Health and Care Sciences, Sahlgrenska Academy, University of Gothenburg, Gothenburg, Sweden; 2 Department of Anaesthesiology, Surgery and Intensive Care Medicine, Sahlgrenska University Hospital, Gothenburg, Sweden; 3 Department of Medicine & Health Sciences, Lund University, Lund, Sweden; 4 Department of Hybride and Interventional procedures, Sahlgrenska University Hospital, Gothenburg, Sweden; 5 Department of Anaesthesiology and Intensive Care, Institution of Clinical Sciences, Sahlgrenska Academy, University of Gothenburg, Gothenburg, Sweden; 6 Centre for Person-Centred Care (GPCC), Sahlgrenska Academy, University of Gothenburg, Gothenburg, Sweden; 7 Faculty of Health Sciences, Department of Nursing and Health Promotion Acute and Critical Illness, Oslo Metropolitan University, Oslo, Norway; 8 Faculty of Nursing and Health Sciences, Nord University, Bodø, Norway; 9 Department of Anesthesiology and Intensive Care, Skåne University Hospital, Lund, Sweden; 10 Department of Anesthesiology and Intensive Care, School of Medical Sciences, Örebro University Hospital, Örebro, Sweden

**Keywords:** Smart glasses, Anaesthesia monitoring, Simulation training, Qualitative research, Anaesthesia care, Patient safety, Vital signs monitoring

## Abstract

**Background:**

Monitoring of vital signs is essential in anaesthesia care and plays a key role in preventing adverse events. Technological innovation is recognised as an important factor in enhancing patient safety. Smart glasses represent a novel tool that may support anaesthesia professionals in monitoring vital signs; however, their practical use and user experiences in anaesthesia care remain insufficiently explored. Understanding anaesthesia healthcare professionals’ experiences with this technology is crucial to ensure its safe and effective implementation. In this study, smart glasses from Microsoft HoloLens 2 were used to visualize vital signs in the user’s field of vision. The aim was to explore anaesthesia health care professionals’ experience of using Microsoft HoloLens 2 smart glasses for monitoring vital signs in various simulated anaesthesia care scenarios.

**Methods:**

A qualitative study design was used to explore the experiences of nurse anaesthetists and anaesthesiologists. Data were gathered through focus group interviews and subsequently analysed using qualitative content analysis.

**Results:**

One overarching theme - a positive yet cautious attitude towards smart glasses - was identified, comprising three categories: Impact on intraoperative monitoring, Usability of the smart glass technology, and Communication challenges. These categories illustrated both advantages and limitations of using smart glasses in simulated anaesthesia care scenarios.

**Conclusions:**

The experience of using Microsoft HoloLens 2 for monitoring vital signs in simulated anaesthesia care scenarios revealed a generally positive but careful attitude towards the technology. Participants appreciated its potential to enhance situational awareness through continuous access to vital signs, while also highlighting concerns related to ergonomics, restricted field of view, and possible distraction. The findings offer insights for developers aiming to optimise smart glasses for clinical use. Further refinement and evaluation in clinical settings are needed before broader implementation in anaesthesia practice.

**Supplementary Information:**

The online version contains supplementary material available at 10.1186/s12871-025-03501-4.

## Background

Patient safety is an important aspect of health care worldwide. During the last 100 years, the specialism of anaesthesia has evolved to become much safer for patients, especially in high income countries [1, 2]. Every day, anaesthesia health care professionals use modern technology and up-to-date checklists and guidelines to be able to deliver high quality care. However, patient harm still occurs, and a majority has been assessed as preventable [3]. Monitoring patients’ vital signs is a crucial aspect of anaesthesia care and has proven important to reduce the risk of accidents and incidents [4]. Innovation and technological progress have historically made anaesthesia safer [5]. Continued improvements in vital sign monitoring is identified as an important factor in strengthening patient safety in future anaesthesia care [1, 2, 6, 7]. One new technology that might improve vital sign monitoring is the use of smart glasses (SG) [8, 9].

SG are produced by various manufacturers and come in a variety of forms and designs. They are worn by the user with a comfort comparable to ordinary glasses. They combine real-world with virtual information and were first developed in the late 1960 s [10]. Modern SG devices incorporate a computer and a miniature camera, enabling functions such as photo capture, video recording, and the visualization of information through a see-through optical display within the user’s field of vision, among other capabilities [11, 12]. SG hold the possibility to share information with other devices, e.g. smartphones, tablets, and computers. They are controlled using voice or touch commands and have emerged as a promising technology in different areas of anaesthesia care during the last decade [13, 14] Health care professionals in anaesthesia see potential in using SG for monitoring vital signs [8, 9, 15]. Identified applications include emergency situations and induction of general anaesthesia [16]. According to Rogers [17], adoption and implementation of a new technology such as SG needs to take place in specific stages to facilitate a process that achieves maximum performance and patient safety. The process starts with “The Knowledge Stage”, where someone learns and seek information about the innovation [17–20]. Although previous studies have investigated the use of SG in anaesthesia care, their application for monitoring vital signs in simulated acute anaesthesia scenarios using Microsoft HoloLens 2 has, to our knowledge, not been explored. For this purpose, the research team in collaboration with software experts developed an application (APP) for Microsoft HoloLens 2 SG designed to visualize vital signs within the user’s field of vision. To ensure the development of a product that is safe and effective for both patients and healthcare personnel, understanding the experiences of intended users is essential. Accordingly, this study aims to generate knowledge regarding the use of Microsoft HoloLens 2 in simulated anaesthesia environments through its implementation in various simulated anaesthesia care scenarios.

### Aim

To explore anaesthesia health care professionals’ experience of using Microsoft HoloLens 2 smart glasses for monitoring vital signs in various simulated anaesthesia care scenarios.

## Methods

The study used a qualitative design within the interpretivist paradigm with focus group interviews. Focus group interviews offer a social interaction between participants which can yield rich, in-depth and meaningful data by exploring different experiences and perspectives [21–23]. This study takes a qualitative approach that conforms to the guidelines for reporting qualitative research [24].

### Participants

The study population comprised nurse anaesthetists, anaesthesiologists in training, and staff anaesthesiologists, all of whom were employed in various operating wards at three hospitals in southern Sweden. Inclusion criteria for participation were that the participants should be nurse anaesthetist, staff anaesthesiologists or anaesthesiologist in training and clinically active within anaesthesia care.

Participants worked at different operation wards specialising in different surgical fields: neurosurgical; ear, nose and throat; plastic surgery; women’s clinic; interventional; cardiac thoracic and orthopaedic patients. Years of experience varied among participants between less than a year to 37 years. Age varied from 28 to 63 years. The selected group is considered relevant for the purpose of addressing the research question.

### Smart glasses

**Fig. 1:**
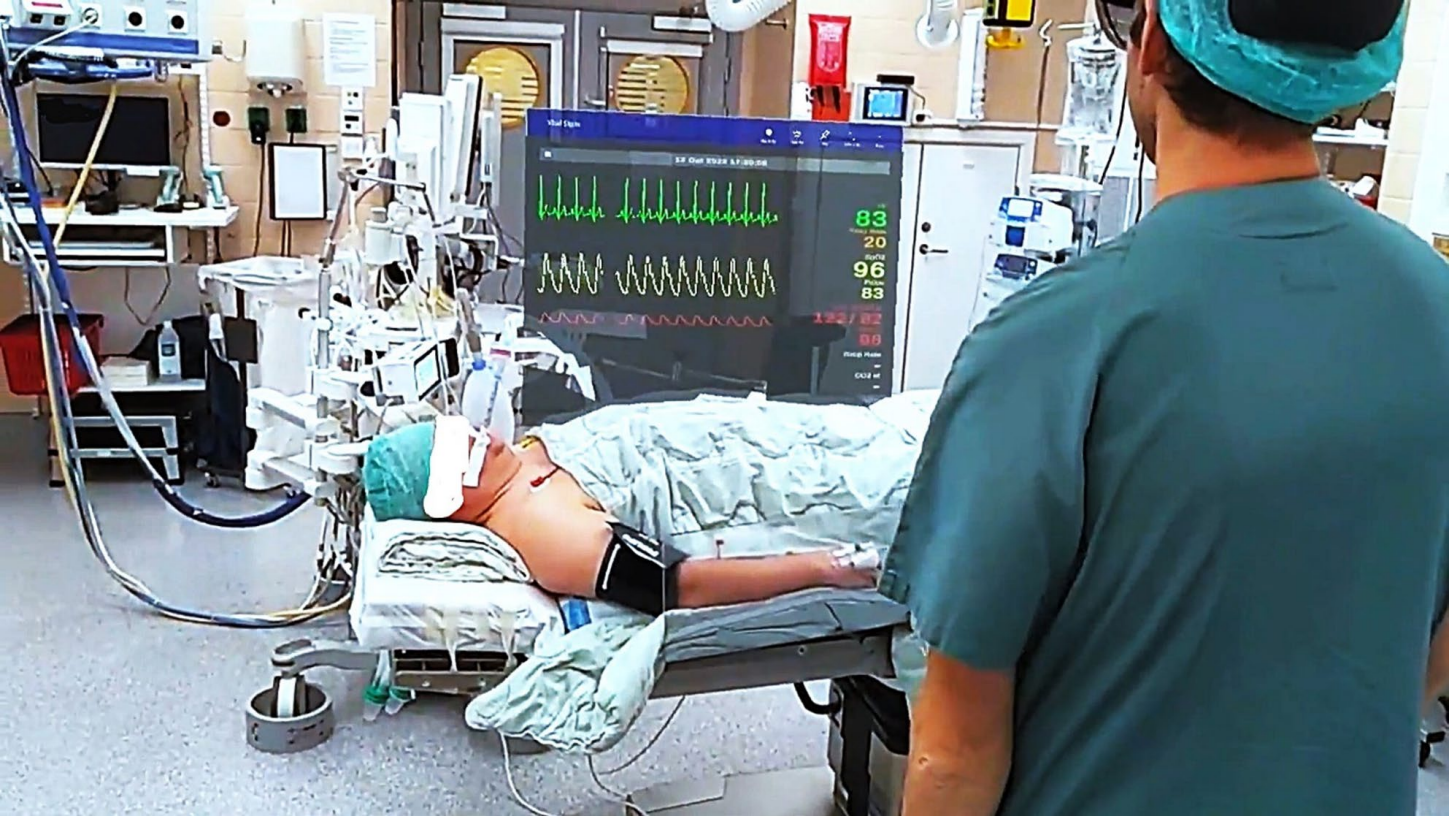
View of vital signs visualized via Microsoft HoloLens 2 using the pin mode

Microsoft HoloLens 2 is an advanced device belonging to mixed reality technology. They combine the real and digital world, making it possible for users to interact and move physical and virtual elements and environments [25]. The vital signs visualized with a numeric value were heart rate, blood oxygenation, blood pressure (non-invasive and invasive), temperature, respiratory rate and ECG. ECG, invasive blood pressure and blood oxygenation were visualized with complementary wave forms. The SG user could choose from four different visualization modes. All of these were shown via SG in the user’s field of vision. The different modes were named pin, heads-up, follow-me and face-me. In *Pin mode* (Fig. 1), the display window was “pinned” to a specific physical location but could be moved or resized by the user to whatever place or size they wished. *Face-me mode* functioned similarly, with the additional feature that the window automatically faced the user when looking at it. In *Heads-up mode*, vital signs remained in the user’s direct line of vision, following every head movement; the window size could be reduced but not repositioned. In *Follow-me mode*, the window followed the user’s head movements but was positioned in the peripheral field of vision. Regardless of mode, the window remained transparent (see- through). To be able to see vital signs in the SG, the product was connected wirelessly to a Next Unit of Computing (NUC). The NUC was connected via a wire to the stationary monitor visualizing vital signs and extracted information from the monitor and send it to the SG.

### Ethical approval

The work was conducted according to the Declaration of Helsinki [26]. Ethical approval was obtained from the ethics review authority, Lund department (dnr 2021 − 01841). Written informed consent was collected from each participant before the simulated scenarios started. Recorded and transcribed material was securely stored on an encrypted USB drive and in a high-security cloud service. All participants remained anonymous, and access to the data was restricted to a limited number of the authors.

### Data collection

SG were used in a simulated environment between February of 2022 and August of 2024. Three simulated scenarios were created based on previously published research [16]. The scenarios were reflecting induction of general anaesthesia, loss of blood pressure during general anaesthesia and cardiac arrest in a patient before starting the induction of general anaesthesia. Each participant used SG in one of these scenarios. The total number of participants in the focus group interviews was 27 (*n* = 27) (Table 1). Prior to entering the study, participants underwent a training session where participants were introduced to the Microsoft HoloLens 2. The training session comprised knowledge on how to start the device, navigate between different applications and menus, and how to access vital signs among other things. Following this introduction, SG were used in various simulated anaesthesiologic scenarios, where participants practiced visualizing vital signs according to the different visualization modes (Table 1). The total training time was either a morning or an afternoon session. Interviews were conducted and audio recorded by the first author using an interview guide (Table 2). Notes were taken during the focus group interviews. The number of participants in each focus group interview varied between two and four. Questions such as “can you give an example?”, “Can you elaborate on what you said?” and “Did I understand you correctly that you mean…?” were asked to follow up some of the answers. During the interviews, also summaries of the participants’ responses were provided to ensure that their statements were accurately understood. Interviews were conducted in a meeting room close to the simulation room immediately after the simulated scenario had concluded. Interviews lasted between 15- and 25-minutes During interviews 7, 8, and 9, only limited new information emerged and the interviewees were beginning to repeat remarks made in previous interviews. This was discussed among the authors, and we agreed that data saturation had been reached [27, 28].

**Table 1:** Demographic data of the focus group interviews for each scenario

	Patient suffering cardiac arrest	Patient suffering loss of blood pressure	Induction of general anaesthesia	
Number of focus groups	3	3	3	*n* = 9
Participants	10	8	9	*n* = 27
Anaesthesiologists	3/10 (30%)	2/8 (25%)	1/9 (11%)	*n* = 6
Nurse anaesthetists	7/10 (70%)	6/8 (75%)	8/9 (89%)	*n* = 21
Female gender	6/10 (60%)	6/8 (75%)	5/9 (56%)	*n* = 17

**Table 2:** To the left. Parts of the interview guide with key questions used during focus group interviews. To the right. Simulated scenarios used during the training session

**Interview guide**	**Training scenarios**
1. How did you experience the SG working in the given situation?	1. Introduction to SG. On/off, log in, how to manage voice and touch command, volume and light settings etc.
2. Would you consider using SG in the same situation in your daily work?	2. Getting used to SG. Try different apps and menus, manage alarms, make changes in setting
3. Tell me about your experiences where you believe SG were helpful.	3. Using SG to monitor a patient’s vital signs during anaesthesia, incl. documentation
4. Tell me about your experiences with risks or problems related to the use of SG	4. Using SG during insertion of an i.v. line.
5. How did you perceive the presentation of information in the glasses?	5. Using SG while administering medications
6. Was there any information you would like to remove from the SG?	6. Using SG while being far away from stationary monitor
7. Was there any information you felt was missing in the SG?	7. Using SG during CT scan
8. How do you think the feeling of control might be affected when using SG?	8. Using SG during MRI
9. How do you think patient safety might be affected when using SG in your daily work (positively or negatively)?	9. Using SG during intra hospital transport
10. Do you have anything else you would like to add regarding your experience from using SG?	10. Using SG during extubating

### Data analysis

Data were analysed using qualitative content analysis as described by Graneheim and Lundman [29]. The focus group interviews were manually transcribed verbatim in Swedish by the first author. The transcripts were then read by PE, CS, MR, and PJ to gain familiarity with the content and obtain a sense of the whole. NVivo 14 (Lumivero, 2023) was used by PE to organize and gain an overview of the analysis process; however, no transcription or analysis was conducted by the software itself. Meaning units (words and sentences) that addressed the aim of the study were identified, condensed and labelled into codes jointly by PE, CS, MR and PJ. Discussions between PE, CS and MR sorted codes into sub-categories, and sub-categories into categories according to their similarities. Similarities and differences were compared to find consensus for the final categories by PE, CS and MR (Table 3). During the interpretation phase the latent content became evident, whereupon one theme was created. The authors involved in the analysis have different preunderstandings regarding SG. PE was well acquainted with SG, and PJ had some knowledge of their use prior to the start of the analysis. CS and MR had no prior knowledge of SG before the analysis began. PE, CS, and PJ possess both theoretical and practical knowledge in anaesthesia, while MR has theoretical and practical knowledge in intensive care.

## Results

In total, 193 min and 59 s of recorded material were transcribed into text, yielding 32,638 words that were analysed. There is an ambivalence regarding the use of SG. While broadly positive, there is a concern about what using the technology will mean for health care professionals within anaesthesia care. Hence the latent content throughout all categories, the theme, is *positive yet cautious attitude towards SG.* Three categories, Impact on intraoperative monitoring, Usability of the smart glass technology and Communication challenges, together with six subcategories, all describe how participants express positive and negative effects of wearing SG (Table 3).

**Table 3:** Overview of the analysis process

Meaning unit	Condensed meaning unit	Code	Sub-category	Category	Theme
You can see the vital signs all the time, like in real time you know, you didn’t have to look for the monitor.	You can see the vital signs all the time without looking for the monitor.	See vital signs all the time.	New possibilities with monitoring vital signs.		
I can’t see that I would experience it (SG) as a better alternative than to perform an intubation with a decent clear view of a regular monitor screen.	Not a better alternative during intubation than a regular monitoring screen.	Did not contribute.	Challenges and limitations for use in various scenarios.	Impact on intraoperative monitoring.	
I like to have a clear view, to be able to see everything and that gets a bit hard when using them (SG).	Harder to get a clear view and see everything with SG.	Affects line of sight.	Usability of wearing the physical glasses.		Positive yet cautious attitude towards smart glasses
I thought it was very good that it (vital sign layout) was similar to the Philips monitor; it was good because then I know where to look to find the blood pressure, it has the same colour, that was good.	Good that vital sign layout was similar to the Philips monitor.	Good layout.	Usability of viewing information.	Usability of the smart glass technology.	
And what I was missing in this situation was to be able to have eye contact with the patient.	Missed having eye contact with the patient in the situation.	Reduced eye contact.	Eye contact possibilities are affected.		
And then you have these monitors in front of you, then it is like, who do you talk to?	Hard to know who you talk to when monitors are in front of you.	Hard to communicate.	Risk of confusing communication.	Communication challenges.	

The result related to each scenario is presented below. Demographic data can be seen in Table 1 and participant characteristics in Table 4.

**Table 4:** Participants characteristics of the focus group interviews divided by scenario

	Patient suffering cardiac arrest	Patient suffering loss of blood pressure	Induction of general anaesthesia
Age, median (range)	35 (32–50)	34.5 (28–63)	38 (29–51)
Years of experience, median (range)	3 (1–37)	3.5 (1–17)	4 (0.5–10)

### Impact on intraoperative monitoring

#### New possibilities with monitoring vital signs

While accessing vital signs wherever you are, it was easier to be physically closer to the patient and still monitor vital signs.

- *It was very comforting when you walked away from the patient to handle medications*,* that you could see everything all the time….* (Participant 9, loss of blood pressure).

During a cardiac arrest, the potential use of SG depended on what kind of task participants were performing during the cardiopulmonary resuscitation (CPR). The person in the leading role thought SG made them get a better overall view of the situation.

- *I had the possibility to be team leader from where I was standing. It was very comfortable to have all the vital signs in front of your eyes; it facilitated the situation.* (Participant 3, cardiac arrest).

All participants, in all three scenarios, believed that SG could be used during intra-hospital transport as well as for extubating and intubation. Familiarity with SG was mentioned to facilitate their use even further, regardless of the scenario they are used in. During cardiac arrest participants believed that it was better to get to know the SG in a calm/elective situation first, before using them in an emergency event.

- *If you practise a lot in calm situations where you use SG*,* then it would be something completely different when you use them in emergency situations.* (Participant 19, cardiac arrest).

The general opinion during induction of general anaesthesia was that SG functioned well and facilitated the work. They were seen as flexible and efficient and less obstructive than expected. Participants believed that the general interest in technology might play a part in the adoption of SG.

- *I think it is important if you think it is fun with new technology. I believe that can be crucial in whether you want to learn or not.* (Participant 22, induction of general anaesthesia).

In all three scenarios, SG improved access to vital signs which was expressed to enhance the possibility to identify changes in vital signs in an early stage.

#### Challenges and limitations for use in various scenarios

During the scenario with loss of blood pressure participants found SG redundant and that the use could be potentially confusing. This may distract the user during patient care, particularly when the software malfunctions. Additionally, SG could affect the feeling of control, because of diminished overview, and some users experienced a sense of claustrophobia.

The ability to handle and manoeuvre SG became evidently harder during cardiac arrest, especially when performing CPR. Participants also indicated that during chest compressions, the display of vital signs provided little added value, and the use of SG was perceived as distracting in the emergency context.

- *At the same time*,* I believe that it will be a disturbing moment. They (vital signs) are in front of you*,* and you get the alarms that you need to silence; I mean*,* it is a situation where you need to keep track of a lot of things.* (Participant 19 cardiac arrest).

During the induction of general anaesthesia, participants expressed concerns that SG could be obstructive and distracting, potentially reducing their sense of control. They also noted that the continuous display of vital signs within the field of vision might inadvertently shift the user’s focus from the patient to the monitor.

- *It might be that you focus more on the screen that was brought up via the glasses then watching the patient that much.* (Participant 17, loss of blood pressure).

For SG to be feasible in future anaesthesia care, both hardware and software improvements are required, including smaller device design and optimized information display placement.

The fact that SG might create a distraction and was redundant became evident in all three scenarios.

### Usability of the smart glass technology

#### Usability of wearing the physical glasses

Regardless of scenario, SG contributed to a darker, more tinted, field of vision which was seen as disturbing. In short-term use, the physical strain on the user’s head and neck was perceived as much lower than with prolonged use of SG. It was also evident in all three scenarios that SG limited the field of vision.

- *There was this little blind spot on the side of the glasses. It got harder to see. It got darker in one direction*,* so you didn’t see the periphery as good as you used to…* (Participant 27, loss of blood pressure).

When participants used SG during cardiac arrest, they thought SG were a bit clumsy, caused a slight pressure to the forehead and put a strain on the neck and eyes of the user. Suggestions were made that the use of SG would be easier if they were lighter and more flexible.

- *I felt it was hard for the neck*,* maybe it was because I was performing chest compressions*,* but I felt something quite early.* (Participant 18, cardiac arrest).

During induction of general anaesthesia SG felt hot to wear from time to time. While some believed that the glasses were more user friendly and that the limitations in the field of vision were less evident than expected others perceived that SG made it harder to visualize chest movements.

- *When you are the person intubating*,* I perceived that it (SG) hinders me from seeing what I want to see*,* it is a bit tinted*,* it hindered me from seeing whether the chest was moving or not.* (Participant 21 induction of general anaesthesia).

#### Usability of viewing information

Discussions in all three scenarios related to the presentation of vital signs in SG. During loss of blood pressure, participants believed that vital signs presented in the heads-up mode came too close and that the mode was sometimes hard to handle. On the other hand, participants also mentioned that the pin mode was something they appreciated and was easy to handle.

- *… I went from pin to heads-up mode. Then the information came really*,* really*,* close*,* and then you have a problem because then you need to change the layout*,* so it became smaller.* (Participant 11).

Participants from all scenarios were pleased with the layout and presentation of vital signs. They were presented with the same colours, font, size, etc. as the Philips stationary monitor used in clinical work. If given the choice, participants wanted to add information from the anaesthesia machine to the current presentation and most participants wanted EtCO2 as a complement to the other vital signs. However, there was a concern that if we were to add more information to existing layout, it might be overwhelming.

- *… I think it might be hard to fit all the curves and all the different pressures from both the anaesthesia machine and the Philips monitor; it might be messy. At the same time*,* you want all the information available.* (Participant 17, loss of blood pressure).

There were several functions that participants wanted to add to the SG during cardiac arrest. These included the ability to see information from the defibrillator, access to treatment guidelines and automatic documentation on a digital journal.

It was suggested that it would be good to have different types of layouts depending on the situation and that the settings for the different modes and layouts should be individual.

- *It would be something if you had a TC (tumour cerebri) mode for monitoring a patient during TC surgery or CPR mode for a CPR situation and maybe you could add the defibrillator.* (Participant 24, cardiac arrest).

Participants experienced technical issues, such as latency in the information and bugs in the application with SG during induction of general anaesthesia and cardiac arrest. Several participants found the issues so disturbing they wanted to remove the SG. It was also perceived as distracting to control SG using English language.

- *It was technical issues*,* so I just ignored them I just wanted to take them off.* (Participant 23, cardiac arrest).

Participants wanted vital signs displayed more peripherally, often in the upper right corner. They suggested adjusting the visualisation of information based on the most important vital signs and preferred a smaller screen with fewer curves.

### Communication challenges

#### Eye contact possibilities are affected

Participants using SG during induction of general anaesthesia believed that it could be harder to build trust between patient and care giver, if you are unable to make eye contact.

- *One thing I thought about is that you can’t build the same trust if you can’t see each other’s eyes.* (Participant 21).

Participants across all scenarios noted that reading body language was impaired. They emphasized that a large part of colleague communication is nonverbal, and that the use of SG reduces the ability to observe eye contact and facial expressions.

#### Risk of confusing communication

Consistent with the other scenarios, participants indicated that using SG during loss of blood pressure may hinder effective communication among anaesthesia colleagues and potentially distract other healthcare professionals in the operating room.

- *If I shout out voice commands*,* that might disturb others in the OR.* (Participant 27, loss of blood pressure).

During cardiac arrest, participants expressed concern that communication could become disorganized and confusing if multiple users wore SG simultaneously, particularly in emergency situations. Similarly, participants using SG during the induction of general anaesthesia believed that team communication could be impaired. They noted that the combination of voice and touch commands required to operate SG might further confuse nearby colleagues.

- *There was this one time when you were doing something with SG*,* and I thought that you wanted my attention on something. Had this happened in reality*,* I would definitely think that there was something going on with the patient.* (Participant 13, induction of general anaesthesia).

## Discussion

This study explored anaesthesia health care professionals’ experiences of using SG from Microsoft HoloLens 2 for monitoring vital signs in simulated anaesthesia care scenarios. Similar to earlier research [8, 16], the findings indicate that SG may improve access to vital signs and support continuous monitoring. These findings where particularly evident in scenarios where the stationary monitor is not in direct view, such as during induction of general anaesthesia or loss of blood pressure. At the same time, the results also reveal several challenges and limitations that must be addressed before SG can be considered useful in routine anaesthesia care.

This study can be positioned within what Rogers [17] refers to as “The Knowledge Stage” of the innovation adoption process. Exposure to new technology often leads to both desirable and undesirable consequences, which are difficult to separate at an early stage of implementation [17]. The participants’ experiences in this study reflect such ambivalence, a generally positive but cautious attitude toward the use of SG.

The results show that participants perceived both benefits and drawbacks when using SG. On the positive side, participants appreciated the constant visibility of vital signs, which they perceived as potentially supportive for early detection of changes. This was described as particularly helpful when physical movement or multitasking was required, as it allowed them to remain focused on the patient while maintaining situational awareness. These experiences are in line with previous findings indicating that head-mounted displays can facilitate monitoring and workflow during anaesthesia procedures [8, 16].

However, the results also highlight important limitations. Participants reported a reduced field of vision, visual obstruction, and physical discomfort such as strain on the head and neck. These effects were especially evident during the cardiac arrest scenario, when participants were physically active and exposed to higher cognitive demands. Similar consequences have been reported previously, where Palumbo [25] described comparable ergonomic and visual limitations among HoloLens 2 users. Although future technological advancements may mitigate such drawbacks, the current version of the device still presents notable usability challenges [25].

Distraction emerged as another concern. Some participants found the continuous presence of visual information potentially disruptive, particularly when technical malfunctions occurred or when multiple alarms were triggered simultaneously. Distractions are well-known risk factors in anaesthesia care and may compromise patient safety [30–32]. Additionally, participants perceived that SG affected communication both with colleagues and patients. The headset’s design reduces visibility of facial expressions and eye contact, and the use of touch or voice commands could confuse others in the operating room. This finding aligns with previous studies showing that both verbal and non-verbal communication are essential for patient safety [33–38], and that communication failures are among the leading causes of adverse events in anaesthesia care [34, 35, 37].

Although the participants described potential advantages in terms of access to vital signs, these findings should be interpreted cautiously. The data reflect experiences from a simulated environment, not actual clinical outcomes. Therefore, while the participants perceived that SG could help them detect changes in vital signs earlier, this should be seen as an experienced potential rather than as demonstrated improvement in patient safety or efficiency. Further research in clinical settings is required to evaluate whether such perceived benefits translate into measurable effects.

The study also indicates that familiarity and training may facilitate SG use. Most participants had limited or no prior experience with SG, and despite introductory training, some handling difficulties remained. Previous research has highlighted that adequate training and repeated exposure are essential for users to integrate new technologies effectively into their workflows [25, 30, 39]. Future studies should therefore explore optimal training approaches and evaluate long-term experiences of SG in clinical anaesthesia practice.

### Limitations

There are limitations to this study. The focus group sample size can be considered small. Two out of nine focus group interviews had two participants. It was expected there would be at least three participants in all focus group interviews; however, in two cases, participants withdrew late because of staff shortages at their workplace. There is no clear evidence for how many participants are needed in focus group interviews [40]; research suggests that as few as two participants can be enough to yield rich data [41]. The data gathered during focus group interviews with two participants contributed with interesting and valuable information and our data clearly shows saturation, which indicates that an adequate sample size was reached [38, 42, 43].

Participants were selected based on the number of individuals involved in the simulated scenarios using SG. Moreover, the sample primarily consisted of younger participants. These factors may have introduced bias, as individuals with a greater interest in new technology, and younger people in general, are typically more open to adopting innovative technologies [44]. This can potentially have influenced the results. However, all anaesthesia healthcare professionals work in a high-tech environment [45, 46] and likely have a general interest in technical solutions and innovations.

There was one moderator present during the focus group interviews. It is suggested that there should be an assistant moderator present who can collect notes and observe non-verbal communication such as facial expressions and sighs, especially for a sensitive topic [47]. In this case, the number of participants made it possible for one researcher to moderate the interviews and observe non-verbal communication at the same time. The moderator had experience of conducting focus group interviews, and the topic in question is not considered sensitive.

The design of the study is based on previous research in which smart glasses from Google (Google Glass) were utilized [8, 16]. In the present study, Microsoft HoloLens 2 were employed, which are considerably larger and more cumbersome than Google Glass. This distinction may influence the results in comparison to earlier studies, as differences in size, weight, and ergonomics could affect user perception and comfort.

Another limitation is that we did not systematically record which visualization position (pin, heads-up, follow-me, or face-me) participants used during the simulation. Although the system allowed for adjustments, all participants reported being able to achieve visualization of vital signs. Therefore, while the qualitative data may reflect experiences across different visualization positions, this is unlikely to have affected the overall conclusions regarding usability and perceived benefit.

Qualitative research has the advantage of providing rich, descriptive knowledge about people’s experiences of complex phenomena such as SG. However, the method can be challenging in terms of credibility, as the researcher’s own values and interpretations may influence the result. In addition, the sample sizes are often small and context-specific, which limits the transferability of the findings [48]. Our sample may be considered relatively small, but the results still provide valuable insights for the development of Microsoft HoloLens 2 in an anaesthetic environment. The fact that several researchers, both junior and senior, with different backgrounds and theoretical perspectives, participated in the analysis contributes to maintaining a credibility.

The findings of this study are based on the use of SG in a simulated environment. Although simulation is an effective method for replicating real clinical situations, it does not fully reflect the participation in an actual clinical event. This may affect the transferability of study findings to clinical practice.

The dependability of the study is strengthened by the fact that all interviews were conducted by the same author. Confirmability was enhanced through discussions among authors with different preunderstandings of the phenomenon during the analysis, which helped minimize interpretive bias.

## Conclusion

This study contributes to the growing understanding of how anaesthesia healthcare professionals experience using SG for vital sign monitoring. The findings reveal a positive yet cautious attitude. Participants recognise potential benefits such as the constant visibility of vital signs but also express clear concerns. Concerns included issues of limited field of vision, physical discomfort, impact on communication, and potential user distraction. These aspects affect patient safety negatively and need to be solved before SG can be implemented generally in clinical anaesthesia care. Nonetheless, future technological advancements and enhanced training are expected to resolve many of these challenges. These insights may guide future development and implementation of SG technology in anaesthesia care. It is fair to believe that SG may be useable as a vital sign monitoring device in future anaesthesia care.

## Supplementary Information

Supplementary Material 1.

Supplementary Material 2.

## Data Availability

The datasets used and/or analysed during the current study are available from the corresponding author on reasonable request.

## Abbreviations

SG: Smart glasses
APP: Application
NUC: Next unit of computing
CPR: Cardiopulmonary resuscitation
TC: Tumor cerebri
