# Supplementary material for: Smart glasses for monitoring vital signs in anaesthesia care settings: a qualitative simulation study
Source: BMC Anesthesiol. 2025 Dec 1;25:604. doi: 10.1186/s12871-025-03501-4 (PMC12670854; doi:10.1186/s12871-025-03501-4)
Supplement: Supplementary file 1 — Supplementary Material 1. [file 12871_2025_3501_MOESM1_ESM.docx]

Additional file 1

| **Training scenarios** |  |
| --- | --- |
| 1. | Introduction to SG. On/off, log in, how to manage voice and touch command, volume and light settings etc. |
| 2. | Getting used to SG. Try different apps and menus, manage alarms, make changes in setting |
| 3. | Using SG to monitor a patient’s vital signs during anaesthesia, incl. documentation |
| 4. | Using SG during insertion of an i.v. line. |
| 5. | Using SG while administering medications |
| 6. | Using SG while being far away from stationary monitor |
| 7. | Using SG during CT scan |
| 8. | Using SG during MRI |
| 9. | Using SG during intra hospital transport |
| 10. | Using SG during extubating |

Overview of training scenarios
