# Supplementary material for: Smart glasses for monitoring vital signs in anaesthesia care settings: a qualitative simulation study
Source: BMC Anesthesiol. 2025 Dec 1;25:604. doi: 10.1186/s12871-025-03501-4 (PMC12670854; doi:10.1186/s12871-025-03501-4)
Supplement: Supplementary file 2 — Supplementary Material 2. [file 12871_2025_3501_MOESM2_ESM.docx]

Additional file 2

**Interview guide**

- Welcome, information regarding the interview.

**Aim**

To explore anaesthesia health care professionals’ experience of using smart glasses for monitoring vital signs in various simulated anaesthesia care scenarios

**Key questions**

1. How did you experience the SG working in the given situation?
2. Would you consider using SG in the same situation in your daily work?
3. Tell me about your experiences where you believe SG were helpful.
4. Tell me about your experiences with risks or problems related to the use of SG
5. How did you perceive the presentation of information in the glasses?
6. Was there any information you would like to remove from the SG?
7. Was there any information you felt was missing in the SG?
8. How do you think the feeling of control might be affected when using SG?
9. How do you think patient safety might be affected when using SG in your daily work (positively or negatively)?

**Follow-up questions.**

- Can you give an example?
- Can you elaborate on what you said?
- In what way?
- Can you describe more?
- Did I understand you correctly that you mean…?
- How did it affect you?
- How did you experience that situation?
- What were you thinking in that situation?

**Discussion rules.**

- Do not talk over each other.
- Be honest and say what you think and feel.
- There are no right or wrong answers.
- All thoughts and comments are equally important and valuable.
- Everything said stays within the group.

Interview guide used in FGI.
